# Developmental trajectories of vocal behaviors in common marmosets as a reference framework for neurobehavioral studies

**DOI:** 10.3389/fncir.2026.1821810

**Published:** 2026-06-17

**Authors:** Xincheng Zhao, Takaaki Kaneko, Wanyi Lu, Ken-ichi Inoue, Masayuki Matsumoto, Masahiko Takada

**Affiliations:** 1 Institute for the Evolutionary Origins of Human Behavior, Kyoto University, Inuyama, Japan; 2 Division of Behavioral Development, Department of System Neuroscience, National Institute for Physiological Sciences, National Institutes of Natural Sciences, Okazaki, Japan; 3 Graduate Institute for Advanced Studies, SOKENDAI, Hayama, Japan; 4 Department of Integrative Anatomy, Graduate School of Medical Sciences, Nagoya City University, Nagoya, Japan; 5 Department of Neurology, The University of Osaka Graduate School of Medicine, Suita, Japan

**Keywords:** automated detection, dataset, development, disease model, marmoset, vocalization

## Abstract

Nonhuman primates are essential model animals for understanding brain development and neurodevelopmental disorders. The common marmoset is becoming a key primate species due to its ease of handling and reproductive efficiency, along with increasing research resources, such as an MRI brain atlas of developing marmosets. The present study aims to further support the use of marmosets by providing a detailed description of the developmental trajectories of vocal behaviors with an automated system to detect and analyze vocalizations. We collected 64,736 vocalizations, classified them into 11 vocal types, and observed significant changes in their vocal behaviors from neonates to 12 weeks of age. Several call types, including Trill, Phee, and Cry, peaked around the sixth week before gradually declining. By contrast, calls such as immature Phee, and Twitter were most frequent at birth but decreased steadily, nearly disappearing by 6–8 weeks. In addition, we provide a platform for automatically detecting these vocalizations using artificial neural networks trained on a dataset applicable across various research contexts. Overall, this study not only describes the developmental trajectories of vocal behaviors in a fixed controlled context across developmental stages, but also serves as a reference framework for analyzing marmoset models of developmental disorders.

## 1 Introduction

Nonhuman primates (NHPs) constitute essential animal models in neuroscience because of their close phylogenetic relationship to humans, and among them, the common marmoset (*Callithrix jacchus*) has become an important species (Feng et al., 2020; Miller et al., 2016; Mitchell and Leopold, 2015; Samandra et al., 2022). Marmosets offer several practical advantages, such as easy handling, relatively rapid development, and efficient reproduction (Kishi et al., 2014), which allow them to complement other NHPs like macaques. These advantages have enabled marmosets to serve as a valuable platform for adapting modern molecular technologies, primarily developed in rodents, for use in NHPs. Because such optimization typically requires several iterations and a relatively large number of animals, marmosets provide an efficient bridge across species. Successful advances in marmosets, including calcium imaging (Kondo et al., 2018; Sadakane et al., 2015), the development of cell-type-specific promoters (Matsuzaki et al., 2024; Shinohara et al., 2016), and the production of gene-modified animals (Park et al., 2016; Sasaki et al., 2009; Sato et al., 2016), have in turn facilitated the broader application of these techniques to macaques.

Furthermore, marmosets are a valuable model for studying developmental processes of social and communicative behaviors and their disruption (Phaniraj et al., 2026). Marmosets have a variety of call repertoires (Agamaite et al., 2015) and exhibit complex communication dynamics through their calls (Grijseels et al., 2024; Oren et al., 2024). Increasing research has revealed vocal plasticity during development, in which vocal behaviors are jointly shaped by interactions between internal maturational processes and possible external factors (Biazzi et al., 2025; Gultekin and Hage, 2017, 2018; Gultekin et al., 2021; Qi et al., 2025; Takahashi et al., 2016, 2017). For example, neonate marmosets produce distinctive immature calls, and social feedback from their parents shapes the trajectory of vocal maturation (Hage, 2020). Research on vocal development in marmosets may offer valuable insights into human vocal learning and the emergence of language. In addition, it will become increasingly important to evaluate the developmental trajectories of animal models for developmental disorders. A seminal study has described altered vocal features, along with changes in cortical synaptogenesis, synaptic function, and gene expression in a marmoset model of autistic spectrum disorder induced by valproic acid treatment (Watanabe et al., 2021).

However, there is still a lack of behavioral paradigms and analytical frameworks to evaluate developmental processes in model marmosets. There are growing research resources for marmosets, including databases of gene expression (Kita et al., 2021; Shimogori et al., 2018) and neuronal connectivity (Majka et al., 2020a; Watakabe et al., 2023), as well as a brain atlas of MRI and histology (Hata et al., 2023; Liu et al., 2020; Majka et al., 2020b; Woodward et al., 2018). By contrast, only a few technical platforms for the behavioral domain are available (Kaneko et al., 2024). The detection and classification of vocalizations in NHPs relies primarily on manual annotation (Yano-Nashimoto et al., 2024), and automated methods using artificial neural networks have recently emerged as a viable option (Lamothe et al., 2025; Oikarinen et al., 2019; Turesson et al., 2016; Wu et al., 2024; Zhang et al., 2018). Although such a computerized system would be particularly beneficial for studying marmoset development, as it requires longitudinal observations and analysis of a substantial number of calls, its application remains relatively scarce.

To address this issue, we aimed at establishing an automated system to analyze the vocal behaviors of developing marmosets. In the present study, we observed processes of vocal development over the first three postnatal months in 12 marmosets and manually annotated a total of 13,270 calls to train neural networks that can accurately detect a variety of marmoset calls. Our results provide a reference framework for studying the vocal development in marmosets and, also, for assessing that of their disease models.

## 2 Methods

### 2.1 Animals

Vocal development was recorded in 12 neonatal marmosets (6 males and 6 females) derived from 9 families. All animal care and experimental procedures were approved by the Animal Welfare and Animal Care Committee of the Center for the Evolutionary Origins of Human Behavior at Kyoto University, and were conducted in accordance with the Guidelines for the Care and Use of Nonhuman Primates established by the same institution. Infant marmosets were housed with their parents, without any food or water deprivation. The subject marmosets in the present study served as a control group in a separate study, providing a reference for genetically modified disease model marmosets. The subject animals were injected intravenously through the tail vein on the day of birth or the next day with the variant of AAV9 vector, AAV.CAP-B10 (Goertsen et al., 2022), which incorporates a scrambled miRNA sequence under the control of the CAG promoter.

### 2.2 Vocal recording experiment

**FIGURE 1:**
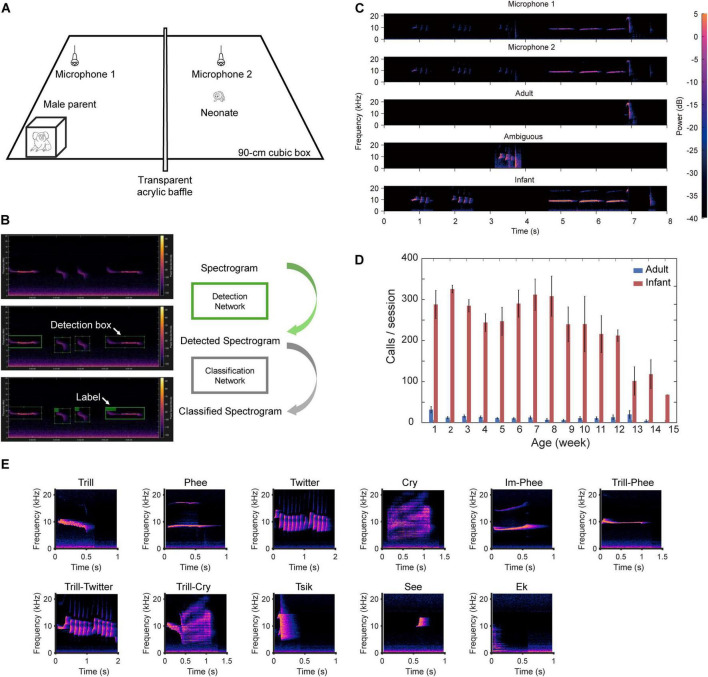
Recording system and analysis framework. **(A)** A schematic illustration of a vocal recording session with two microphones on the ceiling. **(B)** Procedure for the automatic detection and classification of calls. The detection network identified the timing and frequency range of calls; the classification network then classified call types. **(C)** Spectrograms of calls recorded through two microphones, and calls assigned to adult marmosets, ambiguous or infant marmosets. **(D)** Average number of calls per session (±SEM) from adult and infant marmosets. **(E)** Spectrograms of the 11 types of marmoset vocalizations.

The recording booth was a 90-cm cubic box with transparent acrylic walls and a metal mesh floor and ceiling (Figure 1A). The booth was divided into two spaces by a vertically oriented transparent acrylic baffle. Visual, auditory and olfactory contacts were available between animals. Two microphones were placed above the two compartments outside the booth. The male parent was kept in a small metal-mesh cage on the left side of the recording booth. The neonates were gently separated from the parents, kept in a dark area for 1 min, and then placed at the center of the right compartment of the recording booth. The recording duration was 6.5 min. Audio files were recorded at a sampling rate of 44.1 kHz. We recorded vocalizations three sessions per week during the first month of the birth, at least twice per week during the second month, and once per week thereafter up to about 4 months old.

### 2.3 Automated detection system

The neural networks used for vocal analysis consisted of two distinct networks (Figure 1B). The first network detected the timing of calls, irrespective of call types, across all recording sessions, and another network classified call types for each detection. For both networks, we used the analysis system named “DeepSqueak” (Coffey et al., 2019), which was initially developed for the analysis of rodents’ vocalizations and was recently used for the study of primate vocalizations (Romero-Mujalli et al., 2021). The two networks were trained via supervised learning using manually annotated datasets.

Infant and adult calls were discriminated based on the relative call amplitudes recorded by two microphones. A call was attributed to either the infant or the parents when the amplitude difference between the two microphones exceeded a threshold (Figure 1C). Around 5.2% of the calls were identified as the parents’ calls and excluded from the following analysis (Figure 1D).

### 2.4 Datasets for training and quality check of automated detection

A total of 13,270 vocalizations were manually labeled across 45 pseudo-randomly selected recording sessions to comprehensively cover a variety of vocalizations. To construct the training datasets for automated detection, in addition to the 12 marmosets described before, we have additional data from 14 marmosets with gene knockdown, in which a scrambled miRNA sequence in the virus vector was replaced by those for the knockdown of the target gene, such as POGZ (Assia Batzir et al., 2020; Matsumura et al., 2020; Suliman-Lavie et al., 2020) or CHD8 (Katayama et al., 2016; Kawamura et al., 2021). The calls were annotated with timing and frequency ranges on the spectrogram and classified into 11 types (Trill, Phee, Twitter, Cry, Immature Phee (Im-Phee), Trill-Phee, Trill-Twitter, Trill-Cry, Tsik, See, Ek) (Figure 1E).

### 2.5 Statistical test

A two-way ANOVA was performed to evaluate the developmental changes across ages and the differences in call types. The statistical model included the age and call type as main effects, the interaction between these, and subjects as a random factor. The α was set at 0.05.

## 3 Results

### 3.1 Manual annotations for network training

We collected 231 recording sessions over the postnatal 3 months in 12 marmosets to evaluate vocal development. For neural network training, we have annotated 45 sessions (including additional gene-knockdown animals), resulting in 13,270 calls. Among them, 40 sessions with 11,405 calls were used for network training, and the rest were used for performance evaluation of the trained networks (Table 1). The recording sessions used for manual annotations covered most call types; however, two call types (See and Ek) were excluded from later analysis because of small sample sizes.

**TABLE 1:** Number of calls and marmosets annotated for training and quality check datasets.

Dataset type	Class	Trill	Phee	Twitter	Cry	Im-Phee	Trill-Phee	Trill-Twitter	Trill-Cry	Tsik	See	Ek	Noise	Total
Training dataset	Animal	26	25	24	20	20	26	17	11	24	25	9	21	26
	Amount	3012	1236	1283	1643	1786	638	252	81	895	239	77	263	11405
QC dataset	Animal	5	5	5	5	4	4	4	2	5	5	2	2	5
	Amount	346	59	394	276	249	37	132	14	212	55	42	49	1865

### 3.2 Accuracy of the detection network

Manually detected 11,405 calls (Table 1, Training data sets) were utilized to train the detection network. After 24,100 iterations (100 epochs) of training, the detection network achieved a precision of 0.923, a recall of 0.887, and an F1 score of 0.905 A true positive was defined as having an intersection over union (IoU) ≥ 0.5.

### 3.3 Accuracy of the classification network

**FIGURE 2:**
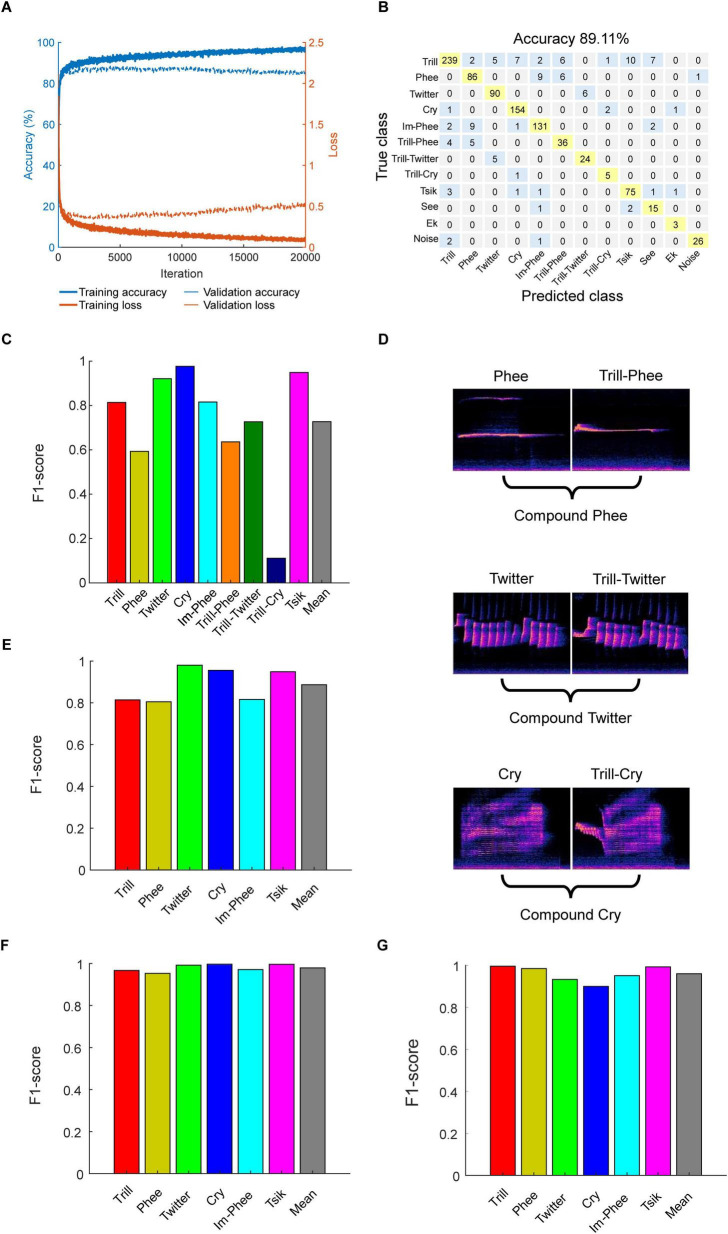
Quality assessment of automated analysis of vocal behaviors. **(A)** Accuracy and loss of the classification network during the training. **(B)** Confusion matrix for the classification network at checkpoint iteration 5,020 with the highest accuracy of 0.891. **(C)** F1 scores for nine call classes [Trill, Phee, Twitter, Cry, Immature Phee (Im-Phee), Trill-Phee, Trill-Twitter, Trill-Cry, Tsik] and the mean. **(D)** Combination of call types. Phee and Trill-Phee were combined into the Compound Phee, as with Twitter and Cry. **(E)** F1 scores for six call classes (Trill, Compound Phee, Compound Twitter, Compound Cry, Im-Phee, Tsik) and the mean. **(F,G)** F1 scores for early **(F)** and later **(G)** developmental stages. Note that these scores were higher than **(E)** as manual annotations were obtained by slightly different procedures (see main text for details).

Manually annotated 11,405 calls (Table 1, Training data sets) were used to train the classification network up to 89,000 iterations (1,000 epochs). During training, 90% of the data were used to update the network weights, and the remaining 10% were used as a validation dataset to determine the optimal number of training iterations. Then, the network state at 5,020 iterations (epoch 57) achieved the highest validation accuracy of 89.1% (Figures 2A,B).

Using a quality-check dataset that is independent of the training dataset, we estimated F1 scores for nine call categories, except for Ek and See, for which there are only a few manual annotation samples (Figure 2C). Some call types showed good classification accuracy, though considerable variation is evident across them. Notably, the confusion matrix (Figure 2B) showed that the classification of compound vocalizations was a significant source of error in the automated detections. For example, there were several erroneous classifications between Trill and Trill-Phee, and between Phee and Trill-Phee (similarly for Cry and Twitter). The F1 scores of all compound calls were lower, particularly for Trill-Cry, which had the fewest samples in the training dataset. We classified the calls into 11 discrete categories. However, there were several complex combinations of acoustic motifs, and there was continuity across calls in different classes. These facts might require an alternative approach to classification based on the definitions of human observers (also see section “4. Discussion”).

To provide a practical and straightforward solution to evaluate developmental trajectories along with the current approach, we combined compound calls with their corresponding individual calls into the same categories (Figure 2D). Then, we simplified the call types to six categories, and obtained F1 scores for each call type (Figure 2E). Overall F1 scores for all call types were over 0.8, ensuring objective and precise evaluation for longitudinal observations for developing marmosets.

To further assess the reliability across developmental stages, we randomly selected 7 sessions from the first 5 weeks (early stage) and the last 5 weeks (later stage), and obtained an F1 score for each group. The detection network extracted unclassified calls from these sessions. Then, these calls were manually classified into call types and compared with the output of the classification network. The results indicated that the classification performance was comparable among age groups, suggesting no systematic bias across developmental stages (Figures 2F,G).

### 3.4 Developmental trajectories as assessed by automated detection

**FIGURE 3:**
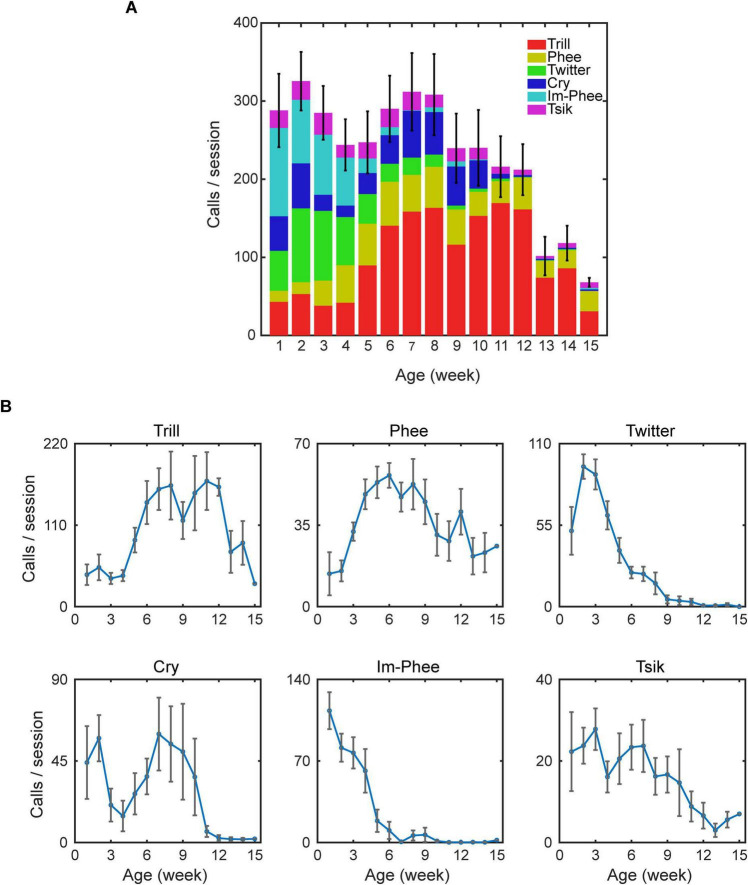
Developmental trajectory of marmoset calls. **(A)** Overall call distribution. Population average (number of calls ± SEM per session) as a function of age (week) for 12 marmoset infants. **(B)** Developmental trajectories (population mean ± SEM) of 6 call types (Trill, Phee, Twitter, Cry, Im-Phee, Tsik) as a function of age.

We then analyzed 231 recording sessions from 12 marmosets to quantify vocal development over 3 months, and the networks identified 64,736 calls. With advancing age, the overall number of vocal behaviors decreased over 15 weeks (Figure 3A) [the main effect of age, *F* (13,715) = 5.31, *p* < 0.0001]. A distinctive pattern of developmental trajectories has been observed in different calls [the interaction between age and call type, *F* (65,715) = 4.63, *p* < 0.0001]. The contact calls, including Phee, Twitter, and Trill, showed similar unimodal distributions, although the time-to-peak varied across call types: Phee and Trill reached peak calling in the 6th–7th week; thereafter, Phee decreased, whereas Trill maintained its peak for another 5 weeks. Twitter reached its peak in the 2nd week, much earlier than the others. The alarm call (Tsik) monotonically decreased over time. Calls typical of neonates showed a different tendency: Im-Phee bottomed out in the 6th week (while Phee reached its peak); Cry exhibited a bimodal pattern, with peaks at weeks 2 and 7, ultimately approaching a negligible level (Figure 3B).

## 4 Discussion

This study aims to establish an efficient and user-friendly method for automated analysis of the development in vocal behaviors of marmosets. We successfully detected marmoset vocalization patterns using DeepSqueak, originally designed for detecting rodent ultrasonic vocal, and all procedures were available via the GUI without modifying the original code. Most calls were detected with high accuracy, except compound calls, which were difficult to distinguish due to their small sample sizes and acoustic similarity. Therefore, we combined the compound calls with their corresponding individual calls (see Figure 2E), thereby improving the reliability of the detection results. Under these conditions, we could elucidate the developmental changes in six different call types. The calls detected by the networks were sufficiently precise, and overall developmental patterns were consistent with those reported in previous studies by manual annotations (Agamaite et al., 2015; Gultekin et al., 2021; Takahashi et al., 2015; Yano-Nashimoto et al., 2024).

Alterations in call distribution may reflect the developmental changes of two distinct but complementary aspects. The first change is the infant affective state during the recording sessions. Infant marmosets spend most of their time being carried by their parents until 4–6 weeks of age, and begin locomoting independently as they grow (Schultz-Darken et al., 2016). This transition is not entirely voluntary but is also shaped by parental behavior, as parents may refuse to carry the infant even when it attempts to climb onto their backs. This process may induce separation anxiety and evoke more calls. Consistent with this view, Cry call, typically used to attract parental attention or elicit parental approach (Ziegler et al., 2017), reached another peak at this age. Subsequently, infants may become less anxious as they get older. Such a developmental change in the affective state was also likely reflected in alarm calls (i.e., Tsik) of which acoustic parameters remained largely invariant across ages, and the frequency of these calls progressively diminished (see Figure 3B). The second change is the transition from immature to mature calls, as evidenced by the inverse trend in Im-Phee and Phee (see Figure 3B). This transition may reflect the physical development of organs involved in vocal production, such as lung and larynx (Takahashi et al., 2015).

It should be noted here that the infant vocalization pattern for some call types may change depending on the experimental protocol in laboratory environments. For example, the increase in Trill calls is prominent in our study during 6–12 weeks, while it is moderate (Yano-Nashimoto et al., 2024) or absent in other studies (Pistorio et al., 2006; Qi et al., 2025; Takahashi et al., 2015). These differences can partially be explained by visual contact between animals. Trill is a contact call used at relatively close distances between animals (Landman et al., 2020), whereas most studies about infant vocal recordings do not allow visual contact with the parents. Similarly, the second peak of Cry calls after 5 weeks was observed in some studies (Gultekin et al., 2021; Yano-Nashimoto et al., 2024) as well as in ours, whereas no such transient increases were observed in others (Qi et al., 2025; Takahashi et al., 2015). It is unclear what experimental parameters might cause these differences, as various studies employ slightly different protocols. Even using the same protocol, individual differences in Cry are high around this age, and, therefore, some parameters in some experimental protocols may exaggerate individual differences arising from the developmental plasticity (Gultekin et al., 2021; Yano-Nashimoto et al., 2024).

The developmental trajectories of neonatal marmoset calls obtained from the automated detection pipeline provide a reference for addressing the developmental process of disease model animals. Impaired language ability often occurs in patients with neurodevelopmental disorders. For example, in patients with White-Sutton syndrome, a recently identified genetic disorder with a wide spectrum of neurocognitive symptoms, speech delay is one of the most universal symptoms (Assia Batzir et al., 2020). The marmoset is an ideal translational model for studying the underlying brain mechanisms (Miller et al., 2016; Samandra et al., 2022; Schultz-Darken et al., 2016). The substantial number of annotations and high analytical precision achieved in the present study offer an efficient framework and a reliable reference for evaluating the vocal development in disease model marmosets.

There are three aspects that should be considered when using the present protocol to evaluate the developmental trajectories of model animals. First, as argued above, the developmental changes in call behavior might reflect multiple components, such as motor development or affective responses to the experimental condition. Thus, interpretations of the behavioral phenotype of model animals should require careful assessment of the mechanisms underlying the behavioral changes. Second, the networks obtained in the present study were optimized for infants or juveniles under 6 months of age, but not for adults. Last, while the present study classified calls into discrete categories, this approach is still insufficient to fully describe the development in marmoset vocal behaviors. Boundaries between calls in different categories may be ambiguous, as shown in Figure 2. On the other hand, the calls within an identical category have variations in their acoustic parameters (Agamaite et al., 2015; Pistorio et al., 2006). A desirable approach could evaluate such continuity over categories and variations within single categories. Recent progress in generative artificial intelligence might provide an alternative approach, such as a Variational Autoencoder (VAE), that is able to capture the complex nature of calls by using a few parameters, thereby enabling quantitative analysis of subtle changes across similar calls (Agamaite et al., 2015), and is used, for example, to assess an atypical vocal development in a rat model of schizophrenia (Potasiewicz et al., 2025). Application of such an approach to the NHP model is worth addressing in a future study.

## Data Availability

The datasets presented in the study are deposited in a public data repository, Zenodo: https://doi.org/10.5281/zenodo.20317624.

## References

[B1] Agamaite J. A. Chang C. J. Osmanski M. S. Wang X. (2015). A quantitative acoustic analysis of the vocal repertoire of the common marmoset (*Callithrix jacchus*). *J. Acoust. Soc. Am.* 138 2906–2928. 10.1121/1.4934268 26627765 PMC4644241

[B2] Assia Batzir N. Posey J. E. Song X. Akdemir Z. C. Rosenfeld J. A. Brown C. W. et al. (2020). Phenotypic expansion of *POGZ*-related intellectual disability syndrome (White-Sutton syndrome). *Am. J. Med. Genet. A* 182 38–52. 10.1002/ajmg.a.61380 31782611 PMC7713511

[B3] Biazzi R. B. Takahashi D. Y. Ghazanfar A. A. (2025). Altricial brains and the evolution of infant vocal learning. *Proc. Natl. Acad. Sci. U. S. A.* 122:e2421095122. 10.1101/2024.10.29.620895 40828014 PMC12403011

[B4] Coffey K. R. Marx R. E. Neumaier J. F. (2019). DeepSqueak: A deep learning-based system for detection and analysis of ultrasonic vocalizations. *Neuropsychopharmacology* 44 859–868. 10.1038/s41386-018-0303-6 30610191 PMC6461910

[B5] Feng G. Jensen F. E. Greely H. T. Okano H. Treue S. Roberts A. C. et al. (2020). Opportunities and limitations of genetically modified nonhuman primate models for neuroscience research. *Proc. Natl. Acad. Sci. U. S. A.* 117 24022–24031. 10.1073/pnas.2006515117 32817435 PMC7533691

[B6] Goertsen D. Flytzanis N. C. Goeden N. Chuapoco M. R. Cummins A. Chen Y. et al. (2022). AAV capsid variants with brain-wide transgene expression and decreased liver targeting after intravenous delivery in mouse and marmoset. *Nat. Neurosci.* 25 106–115. 10.1038/s41593-021-00969-4 34887588

[B7] Grijseels D. M. Fairbank D. A. Miller C. T. (2024). A model of marmoset monkey vocal turn-taking. *Proc. R. Soc. B Biol. Sci.* 291:20240150. 10.1098/rspb.2024.0150 38955229 PMC11334984

[B8] Gultekin Y. B. Hage S. R. (2017). Limiting parental feedback disrupts vocal development in marmoset monkeys. *Nat. Commun.* 8:14046. 10.1038/ncomms14046 28090084 PMC5241798

[B9] Gultekin Y. B. Hage S. R. (2018). Limiting parental interaction during vocal development affects acoustic call structure in marmoset monkeys. *Sci. Adv.* 4:eaar4012. 10.1126/sciadv.aar4012 29651461 PMC5895450

[B10] Gultekin Y. B. Hildebrand D. G. C. Hammerschmidt K. Hage S. R. (2021). High plasticity in marmoset monkey vocal development from infancy to adulthood. *Sci. Adv.* 7:eabf2938. 10.1126/sciadv.abf2938 34193413 PMC8245035

[B11] Hage S. R. (2020). The role of auditory feedback on vocal pattern generation in marmoset monkeys. *Curr. Opin. Neurobiol.* 60 92–98. 10.1016/j.conb.2019.10.011 31835132

[B12] Hata J. Nakae K. Tsukada H. Woodward A. Haga Y. Iida M. et al. (2023). Multi-modal brain magnetic resonance imaging database covering marmosets with a wide age range. *Sci. Data* 10:221. 10.1038/s41597-023-02121-2 37105968 PMC10250358

[B13] Kaneko T. Matsumoto J. Lu W. Zhao X. Ueno-Nigh L. R. Oishi T. et al. (2024). Deciphering social traits and pathophysiological conditions from natural behaviors in common marmosets. *Curr. Biol.* 34 2854–2867.e5. 10.1016/j.cub.2024.05.033 38889723

[B14] Katayama Y. Nishiyama M. Shoji H. Ohkawa Y. Kawamura A. Sato T. et al. (2016). CHD8 haploinsufficiency results in autistic-like phenotypes in mice. *Nature* 537 675–679. 10.1038/nature19357 27602517

[B15] Kawamura A. Katayama Y. Kakegawa W. Ino D. Nishiyama M. Yuzaki M. et al. (2021). The autism-associated protein CHD8 is required for cerebellar development and motor function. *Cell Rep.* 35:108932. 10.1016/j.celrep.2021.108932 33826902

[B16] Kishi N. Sato K. Sasaki E. Okano H. (2014). Common marmoset as a new model animal for neuroscience research and genome editing technology. *Dev. Growth Differ.* 56 53–62. 10.1111/dgd.12109 24387631

[B17] Kita Y. Nishibe H. Wang Y. Hashikawa T. Kikuchi S. S. U M. et al. (2021). Cellular-resolution gene expression profiling in the neonatal marmoset brain reveals dynamic species- and region-specific differences. *Proc. Natl. Acad. Sci. U. S. A.* 118:e2020125118. 10.1073/pnas.2020125118 33903237 PMC8106353

[B18] Kondo T. Saito R. Otaka M. Yoshino-Saito K. Yamanaka A. Yamamori T. et al. (2018). Calcium transient dynamics of neural ensembles in the primary motor cortex of naturally behaving monkeys. *Cell Rep.* 24 2191–2195.e4. 10.1016/j.celrep.2018.07.057 30134178

[B19] Lamothe C. Obliger-Debouche M. Best P. Trapeau R. Ravel S. Artières T. et al. (2025). A large annotated dataset of vocalizations by common marmosets. *Sci. Data* 12:782. 10.1038/s41597-025-04951-8 40360502 PMC12075697

[B20] Landman R. Sharma J. Hyman J. B. Fanucci-Kiss A. Meisner O. Parmar S. et al. (2020). Close-range vocal interaction in the common marmoset (*Callithrix jacchus*). *PLoS One* 15:e0227392. 10.1371/journal.pone.0227392 32298305 PMC7161973

[B21] Liu C. Ye F. Q. Newman J. D. Szczupak D. Tian X. Yen C. C.-C. et al. (2020). A resource for the detailed 3D mapping of white matter pathways in the marmoset brain. *Nat. Neurosci.* 23 271–280. 10.1038/s41593-019-0575-0 31932765 PMC7007400

[B22] Majka P. Bai S. Bakola S. Bednarek S. Chan J. M. Jermakow N. et al. (2020a). Open access resource for cellular-resolution analyses of corticocortical connectivity in the marmoset monkey. *Nat. Commun.* 11:1133. 10.1038/s41467-020-14858-0 32111833 PMC7048793

[B23] Majka P. Bednarek S. Chan J. M. Jermakow N. Liu C. Saworska G. et al. (2020b). Histology-based average template of the marmoset cortex with probabilistic localization of cytoarchitectural areas. *Neuroimage* 226:117625. 10.1016/j.neuroimage.2020.117625 33301940

[B24] Matsumura K. Seiriki K. Okada S. Nagase M. Ayabe S. Yamada I. et al. (2020). Pathogenic POGZ mutation causes impaired cortical development and reversible autism-like phenotypes. *Nat. Commun.* 11:859. 10.1038/s41467-020-14697-z 32103003 PMC7044294

[B25] Matsuzaki Y. Fukai Y. Konno A. Hirai H. (2024). Optimal different adeno-associated virus capsid/promoter combinations to target specific cell types in the common marmoset cerebral cortex. *Mol. Ther. Methods Clin. Dev.* 32:101337. 10.1016/j.omtm.2024.101337 39391837 PMC11466621

[B26] Miller C. T. Freiwald W. A. Leopold D. A. Mitchell J. F. Silva A. C. Wang X. (2016). Marmosets: A neuroscientific model of human social behavior. *Neuron* 90 219–233. 10.1016/j.neuron.2016.03.018 27100195 PMC4840471

[B27] Mitchell J. F. Leopold D. A. (2015). The marmoset monkey as a model for visual neuroscience. *Neurosci. Res.* 93 20–46. 10.1016/j.neures.2015.01.008 25683292 PMC4408257

[B28] Oikarinen T. Srinivasan K. Meisner O. Hyman J. B. Parmar S. Fanucci-Kiss A. et al. (2019). Deep convolutional network for animal sound classification and source attribution using dual audio recordings. *J. Acoust. Soc. Am.* 145:654. 10.1121/1.5087827 30823820 PMC6786887

[B29] Oren G. Shapira A. Lifshitz R. Vinepinsky E. Cohen R. Fried T. et al. (2024). Vocal labeling of others by nonhuman primates. *Science* 385 996–1003. 10.1126/science.adp3757 39208084

[B30] Park J. E. Zhang X. F. Choi S.-H. Okahara J. Sasaki E. Silva A. C. (2016). Generation of transgenic marmosets expressing genetically encoded calcium indicators. *Sci. Rep.* 6:34931. 10.1038/srep34931 27725685 PMC5057151

[B31] Phaniraj N. Brügger R. K. Cerrito P. Burkart J. M. (2026). Opportunities and mechanisms for learning through social interactions: Lessons from marmosets. *Philos. Trans. R. Soc. B Biol. Sci.* 381:20240367. 10.1098/rstb.2024.0367 41641497

[B32] Pistorio A. L. Vintch B. Wang X. (2006). Acoustic analysis of vocal development in a new world primate, the common marmoset (*Callithrix jacchus*). *J. Acoust. Soc. Am.* 120 1655–1670. 10.1121/1.2225899 17004487

[B33] Potasiewicz A. Mincikiewicz Z. Popik P. Nikiforuk A. (2025). Infant rat ultrasonic vocalizations in the neurodevelopmental model of schizophrenia. *Sci. Rep.* 15:27472. 10.1038/s41598-025-08412-5 40721614 PMC12304202

[B34] Qi R. Lin Y. Liu S. Cao X. Xie M. Yu C. et al. (2025). Vocal taking turns is premature at birth and improved by the postnatal phonetic environment in marmosets. *Natl. Sci. Rev.* 12:nwaf162. 10.1093/nsr/nwaf162 40636103 PMC12239203

[B35] Romero-Mujalli D. Bergmann T. Zimmermann A. Scheumann M. (2021). Utilizing DeepSqueak for automatic detection and classification of mammalian vocalizations: A case study on primate vocalizations. *Sci. Rep.* 11:24463. 10.1038/s41598-021-03941-1 34961788 PMC8712519

[B36] Sadakane O. Masamizu Y. Watakabe A. Terada S.-I. Ohtsuka M. Takaji M. et al. (2015). Long-term two-photon calcium imaging of neuronal populations with subcellular resolution in adult non-human primates. *Cell Rep.* 13 1989–1999. 10.1016/j.celrep.2015.10.050 26655910

[B37] Samandra R. Haque Z. Z. Rosa M. G. P. Mansouri F. A. (2022). The marmoset as a model for investigating the neural basis of social cognition in health and disease. *Neurosci. Biobehav. Rev.* 138:104692. 10.1016/j.neubiorev.2022.104692 35569579

[B38] Sasaki E. Suemizu H. Shimada A. Hanazawa K. Oiwa R. Kamioka M. I et al. (2009). Generation of transgenic non-human primates with germline transmission. *Nature* 459 523–527. 10.1038/nature08090 19478777

[B39] Sato K. Oiwa R. Kumita W. Henry R. Sakuma T. Ito R. et al. (2016). Generation of a nonhuman primate model of severe combined immunodeficiency using highly efficient genome editing. *Cell Stem Cell* 19 127–138. 10.1016/j.stem.2016.06.003 27374787

[B40] Schultz-Darken N. Braun K. M. Emborg M. E. (2016). Neurobehavioral development of common marmoset monkeys. *Dev. Psychobiol.* 58 141–158. 10.1002/dev.21360 26502294 PMC4829073

[B41] Shimogori T. Abe A. Go Y. Hashikawa T. Kishi N. Kikuchi S. S. et al. (2018). Digital gene atlas of neonate common marmoset brain. *Neurosci. Res.* 128 1–13. 10.1016/j.neures.2017.10.009 29111135

[B42] Shinohara Y. Konno A. Takahashi N. Matsuzaki Y. Kishi S. Hirai H. (2016). Viral vector-based dissection of marmoset GFAP promoter in mouse and marmoset brains. *PLoS One* 11:e0162023. 10.1371/journal.pone.0162023 27571575 PMC5003399

[B43] Suliman-Lavie R. Title B. Cohen Y. Hamada N. Tal M. Tal N. et al. (2020). *Pogz* deficiency leads to transcription dysregulation and impaired cerebellar activity underlying autism-like behavior in mice. *Nat. Commun.* 11:5836. 10.1038/s41467-020-19577-0 33203851 PMC7673123

[B44] Takahashi D. Y. Fenley A. R. Ghazanfar A. A. (2016). Early development of turn-taking with parents shapes vocal acoustics in infant marmoset monkeys. *Philos. Trans. R. Soc. B Biol. Sci.* 371:20150370. 10.1098/rstb.2015.0370 27069047 PMC4843608

[B45] Takahashi D. Y. Fenley A. R. Teramoto Y. Narayanan D. Z. Borjon J. I. Holmes P. et al. (2015). Language development. The developmental dynamics of marmoset monkey vocal production. *Science* 349 734–738. 10.1126/science.aab1058 26273055

[B46] Takahashi D. Y. Liao D. A. Ghazanfar A. A. (2017). Vocal learning via social reinforcement by infant marmoset monkeys. *Curr. Biol.* 27 1844–1852.e6. 10.1016/j.cub.2017.05.004 28552359

[B47] Turesson H. K. Ribeiro S. Pereira D. R. Papa J. P. de Albuquerque V. H. C. (2016). Machine learning algorithms for automatic classification of marmoset vocalizations. *PLoS One* 11:e0163041. 10.1371/journal.pone.0163041 27654941 PMC5031457

[B48] Watakabe A. Skibbe H. Nakae K. Abe H. Ichinohe N. Rachmadi M. F. et al. (2023). Local and long-distance organization of prefrontal cortex circuits in the marmoset brain. *Neuron* 111 2258–2273.e10. 10.1016/j.neuron.2023.04.028 37196659 PMC10789578

[B49] Watanabe S. Kurotani T. Oga T. Noguchi J. Isoda R. Nakagami A. et al. (2021). Functional and molecular characterization of a non-human primate model of autism spectrum disorder shows similarity with the human disease. *Nat. Commun.* 12:5388. 10.1038/s41467-021-25487-6 34526497 PMC8443557

[B50] Woodward A. Hashikawa T. Maeda M. Kaneko T. Hikishima K. Iriki A. et al. (2018). The Brain/MINDS 3D digital marmoset brain atlas. *Sci. Data* 5:180009. 10.1038/sdata.2018.9 29437168 PMC5810420

[B51] Wu B. Takamichi S. Sakti S. Nakamura S. (2024). Learning marmoset vocal patterns with a masked autoencoder for robust call segmentation, classification, and caller identification. *arXiv [Preprints]* 10.48550/arXiv.2410.23279

[B52] Yano-Nashimoto S. Truzzi A. Shinozuka K. Murayama A. Y. Kurachi T. Moriya-Ito K. et al. (2024). Anxious about rejection, avoidant of neglect: Infant marmosets tune their attachment based on individual caregiver’s parenting style. *Commun. Biol.* 7:212. 10.1038/s42003-024-05875-6 38378797 PMC10879543

[B53] Zhang Y.-J. Huang J.-F. Gong N. Ling Z.-H. Hu Y. (2018). Automatic detection and classification of marmoset vocalizations using deep and recurrent neural networks. *J. Acoust. Soc. Am.* 144 478–487. 10.1121/1.5047743 30075670

[B54] Ziegler T. E. Sosa M. E. Colman R. J. (2017). Fathering style influences health outcome in common marmoset (*Callithrix jacchus*) offspring. *PLoS One* 12:e0185695. 10.1371/journal.pone.0185695 28957433 PMC5619809

